# Glucose availability controls ATF4-mediated MITF suppression to drive melanoma cell growth

**DOI:** 10.18632/oncotarget.16514

**Published:** 2017-03-23

**Authors:** Jennifer Ferguson, Michael Smith, Isabel Zudaire, Claudia Wellbrock, Imanol Arozarena

**Affiliations:** ^1^ Manchester Cancer Research Centre, Faculty of Biology, Medicine and Health, University of Manchester, M13 9PT, Manchester, UK; ^2^ Navarrabiomed-Fundación Miguel Servet-Idisna, Calle Irunlarrea, 3 Complejo Hospitalario de Navarra, 31008, Pamplona, Spain

**Keywords:** melanoma, glucose, MITF, ATF4, ROS

## Abstract

It is well know that cancer cells have adopted an altered metabolism and that glucose is a major source of energy for these cells. In melanoma, enhanced glucose usage is favoured through the hyper-activated MAPK pathway, which suppresses OXPHOS and stimulates glycolysis. However, it has not been addressed how glucose availability impacts on melanoma specific signaling pathways that drive melanoma cell proliferation. Here we show that melanoma cells are dependent on high glucose levels for efficient growth. Thereby, glucose metabolism controls the expression of the melanoma fate transcription factor MITF, a master regulator of melanoma cell survival and proliferation, invasion and therapy resistance. Restriction of glucose availability to physiological concentrations induces the production of reactive oxygen species (ROS). Increased ROS levels lead to the up-regulation of AFT4, which in turn suppresses MITF expression by competing with CREB, an otherwise potent inducer of the *MITF* promoter. Our data give new insight into the complex regulation of MITF, a key regulator of melanoma biology, and support previous findings that link metabolic disorders such as hyperglycemia and diabetes with increased melanoma risk.

## INTRODUCTION

Cutaneous melanoma is a type of skin cancer that originates from melanocytes, specialised cells residing at the basement membrane of the epidermis. The main role of melanocytes is to protect the skin from the damaging effects of UV radiation through the synthesis and secretion of melanin. At the genetic level melanomas are characterised by the presence of activating mutations in genes regulating the RAS/MAPK pathway. Between 40–50% of human melanomas harbour activating mutations in BRAF, while approximately 15–20% of melanomas possess mutations in the NRAS gene [1]. Overall it is thought that about 90% of human cutaneous melanomas show hyper-activation of the MAPK pathway, which contributes to melanoma initiation and progression to metastatic disease [2].

Throughout progression, the viability of melanoma cells depends on their capacity to adapt to new and challenging environments through a diverse range of survival mechanisms such as autophagy, endoplasmic reticulum stress or metabolic reprogramming. Cancer cells are subject to the Warburg effect, by which cells metabolize elevated amounts of glucose through a high (and inefficient) glycolytic rate at the expense of increased lactate production and lower oxidative phosphorylation (OXPHOS) [3]. The Warburg effect fulfils the energetic (ATP) and biosynthetic requirements of cancer cells. Indeed it is widely accepted that the altered metabolism of cancer cells renders these cells dependent on the availability of glucose throughout tumour progression [3]. In melanoma, enhanced glucose usage is favoured through the hyper-activated MAPK pathway, which suppresses OXPHOS and stimulates glycolysis [4, 5]

The need for high levels of glucose has initiated several clinical trials testing drugs that target glucose metabolism and has been extensively applied for cancer imaging [6–8]. In the body glucose availability is controlled by a balance of gluconeogenesis and glycolysis to maintain the concentration of glucose in blood (and therefore available to each organ of the organism) in a dynamic equilibrium between fasting and postprandial levels of around 0.72–1.1 mg/ml (4–6 mM) and 1.4 mg/ml (7.7 mM) respectively (www.diabetes.co.uk). On the other hand, in diabetic patients fasting levels of glucose are not correctly regulated and concentrations of glucose can reach and surpass 2 mg/ml (11 mM). Notably metabolic related conditions such as diabetes, obesity and hyperglycemia have been linked to higher risk of a variety of cancers (colorectal, breast cancer) but also to melanoma [9–11].

Despite evidence for the crucial role played by glucose as a fuel source for cancer cells and the links between high blood glucose levels and melanoma incidence, little is known about how glucose availability impacts on specific drivers of melanoma progression. Therefore we studied the effect of glucose availability on melanoma cell growth with the aim to gain insight into the relevant signalling mechanisms affected by the dependence of melanoma cells on glucose.

## RESULTS

### Melanoma cells, but not melanocytes use glucose for cell growth

**Figure 1:**
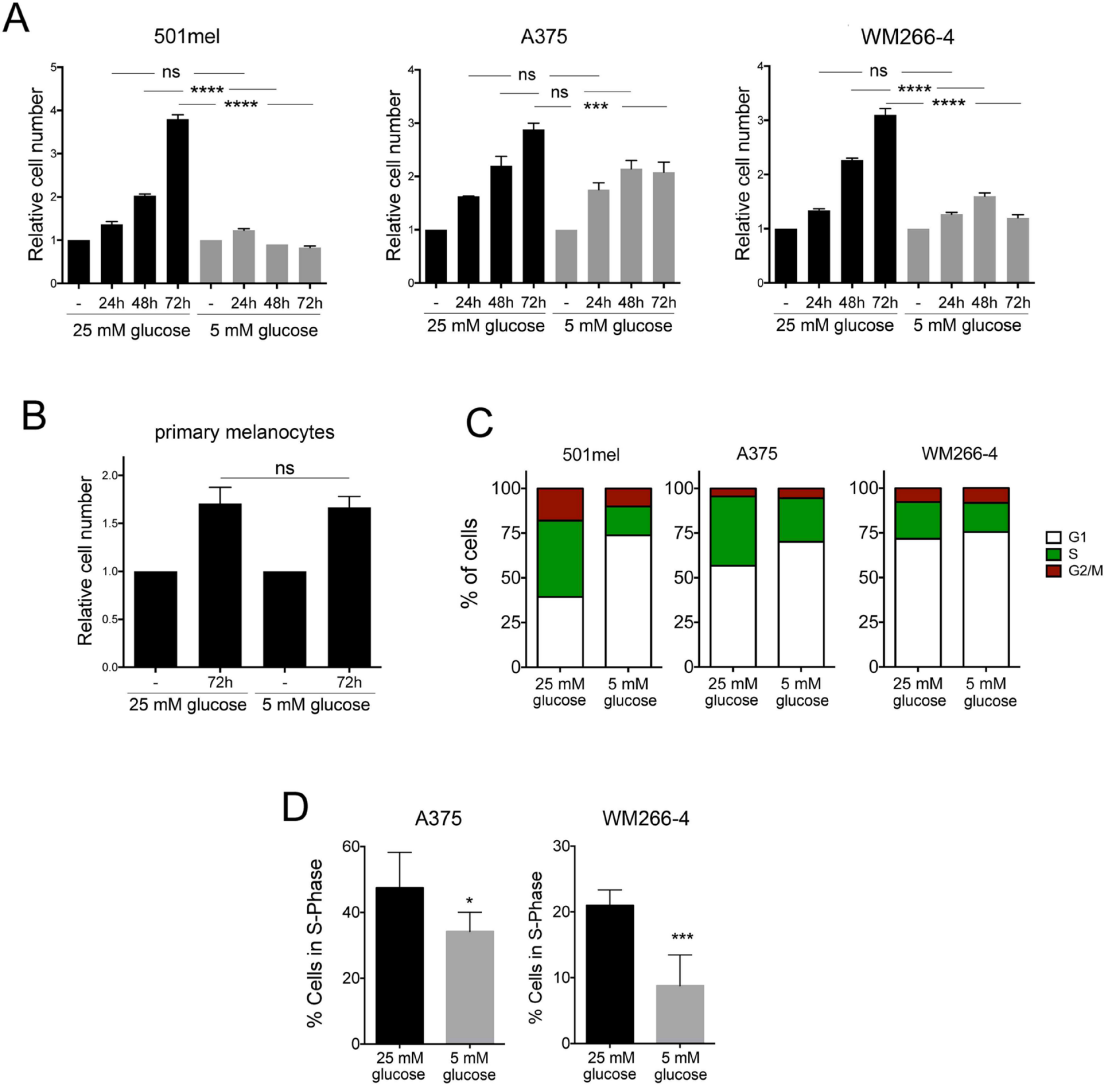
Effect of glucose restriction on melanoma cell proliferation (**A**) WM266-4, 501mel and A375 cells were cultured under high (25 mM) or physiological (5 mM) glucose conditions for 3 days. Cells were counted every 24 h and data is presented as relative proliferation to the number of cells on day 0. (**B**) Human primary melanocytes were cultured at 25 mM or 5 mM glucose and the relative number of cells was assessed after 3 days. One-way Anova was used to assess statistical significance in the relative proliferation of cells treated with different concentrations of glucose over time. (**C**) WM266-4, A375 and 501mel cells cultured for 48 h at 25 mM or 5 mM glucose followed by FACS analysis. The relative distribution in G1, S and G2/M phases of the cell cycle is shown. (**D**) DNA synthesis in S-phase was measured by EdU incorporation into WM266-4 and A375 cells treated as indicated. Student's *t* test was used for statistical comparisons. n.s: not significant, *p < 0.05, ***p < 0.001, ****p < 0.0001.

In order to assess how glucose levels impact on melanoma cell proliferation 501mel (BRAFV600E), A375 (BRAFV600E) and WM266-4 (BRAFV600D) melanoma cells were cultured in the presence of high levels of glucose (25 mM, a concentration normally used for cell culture research) or 5 mM of glucose (physiological fasting concentrations of glucose in human blood). Cell proliferation was analysed by determining the relative cell number compared to day 0 every 24 hours for 3 days (Figure 1A). We observed that reducing glucose concentrations to physiological (5 mM) levels blocked cell growth in all three cell lines when compared with cells grown under high glucose levels, where cell growth was significantly induced (Figure 1A). In contrast, there was no growth stimulatory effect of glucose in primary normal human melanocytes, and the growth rates were independent of glucose availability (Figure 1B). When we assessed individual phases of the cell cycle, we found that reducing glucose levels resulted in an increased number of cells in G1 and a decrease in the proportion of cells in S-phase in all three cell lines (Figure 1C), indicating that melanoma cells use glucose to drive cell cycle progression. Because A375 and WM266-4 cells displayed a weaker effect of glucose levels on cells in S-phase when assessed by FACS analysis, we further confirmed this observation by analysing EdU incorporation. This revealed that glucose restriction induced a significant reduction in the proportion of A375 and WM266-4 in S-phase (Figure 1D).

### High glucose levels regulate melanoma cell cycle progression via MITF

In view of these results we decided to characterize the signalling mechanism by which glucose stimulates melanoma proliferation.

**Figure 2:**
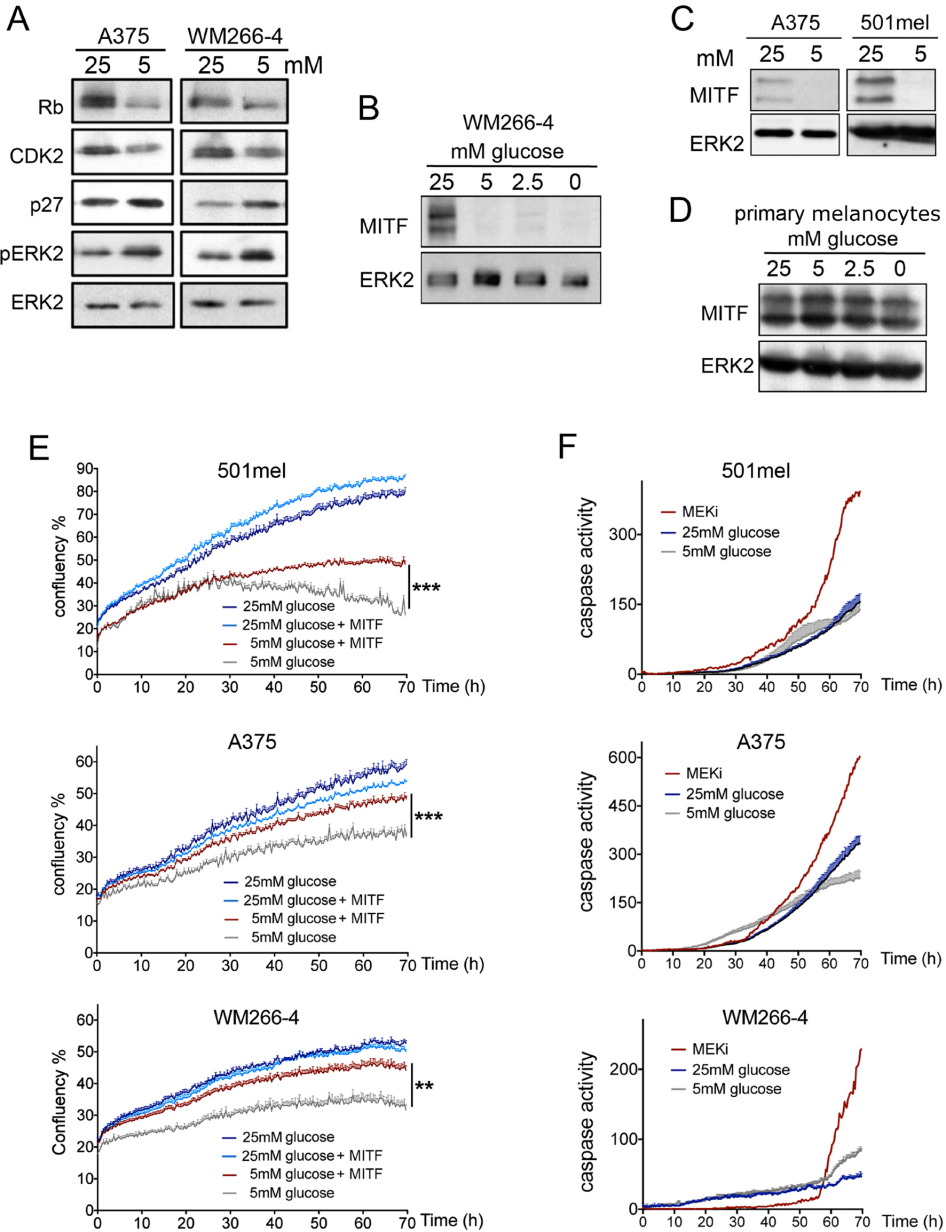
Glucose availability regulates MITF expression in melanoma cells (**A**) Western blot for the expression of Rb, CDK2, p27 and phospho-ERK1/2, in lysates from WM266-4 and A375 cells. (**B**) Western blot for the expression of MITF in WM266-4 cells cultured for 48 h with the indicated concentrations of glucose. (**C**) Western blot for MITF in A375 and 501mel cell lysates after 48 h at 25 mM or 5 mM glucose. (**D**) Western blot for the expression of MITF in primary melanocytes cultured for 48 h with the indicated concentrations of glucose. In all Western blots ERK2 was used as a loading control. (**E**) IncuCyte growth curves measuring cell confluence over time for 501mel, A375 and WM266-4 cells with or without ectopic MITF expression cultured at 25 or 5 mM glucose for 70 h. (**F**) IncuCyte activity curves measuring the accumulation of active caspase 3/7 over time for the indicated cell lines at 25 mM or 5 mM glucose. As positive control for the indication of apoptosis the MEK inhibitor (MEKi) AZD6244 was used at 0.5 μM (WM266-4, A375) or 5 μM (501mel). Student's *t* test was used for statistical comparisons. **p < 0.01, ***p < 0.001.

Due to the central role played by the BRAF/MAPK pathway in melanoma proliferation and cell cycle progression we first assessed if glucose restriction could affect ERK activation. However, glucose deprivation did not inhibit the activation of the MAPK pathway, and even led to an increase in phospho-ERK in A375 and WM266-4 cells (Figure 2A), which could be due to feedback signalling within the pathway. We therefore analysed key cell cycle regulators and observed that after 48 h of glucose deprivation, the shift in the Rb protein, indicating its hyper-phosphorylation was reduced (Figure 2A). This was accompanied by decreased expression of CDK2 and an increase in p27 (Figure 2A). This finding was intriguing as both the CDK2 gene and p27 protein turnover are controlled by the same melanoma cell master regulator, MITF [12, 13]. We therefore tested whether glucose restriction might limit melanoma cell proliferation by affecting MITF expression. As seen in Figure 2B, MITF protein levels were indeed dependent on the availability of glucose in the culture medium, where its expression was regulated in a dose dependent manner (Figure 2B). Similarly, in 501mel and A375 cells glucose restriction induced a profound reduction in the expression of MITF protein (Figure 2C). On the other hand, and in line with the observation that melanocytes do not require glucose for proliferation (see Figure 1B), MITF expression was not regulated by glucose in melanocytes (Figure 2D).

To assess whether there was a causal link between glucose-mediated MITF expression and glucose-dependent growth in melanoma cells, we ectopically expressed MITF from the cytomegalovirus (CMV) promoter [14], which was not affected by glucose levels (Supplementary Figure 1). Continuous assessment of cell growth using the IncuCyte cell growth analysis system revealed that ectopic-MITF expressing cells were significantly more resistant to glucose restriction than their parental counterparts (Figure 2E). We then used the same system to test whether growth inhibition was due to an increase in cell death and/or just an effect on proliferation. As shown in Figure 2F, glucose restriction did not induce a significant increase in caspase-3/7 activation. These results, together with those presented in Figure 1 strongly support the notion that MITF expression is required for glucose mediated cell cycle progression and proliferation in melanoma cells.

Thus, in summary, in melanoma cells, it appears that an altered glucose metabolism has seized control over the expression of MITF, thereby rendering these cells dependent on glucose to drive proliferation.

### In melanoma cells glucose regulates transcription from the *MITF* promoter

**Figure 3:**
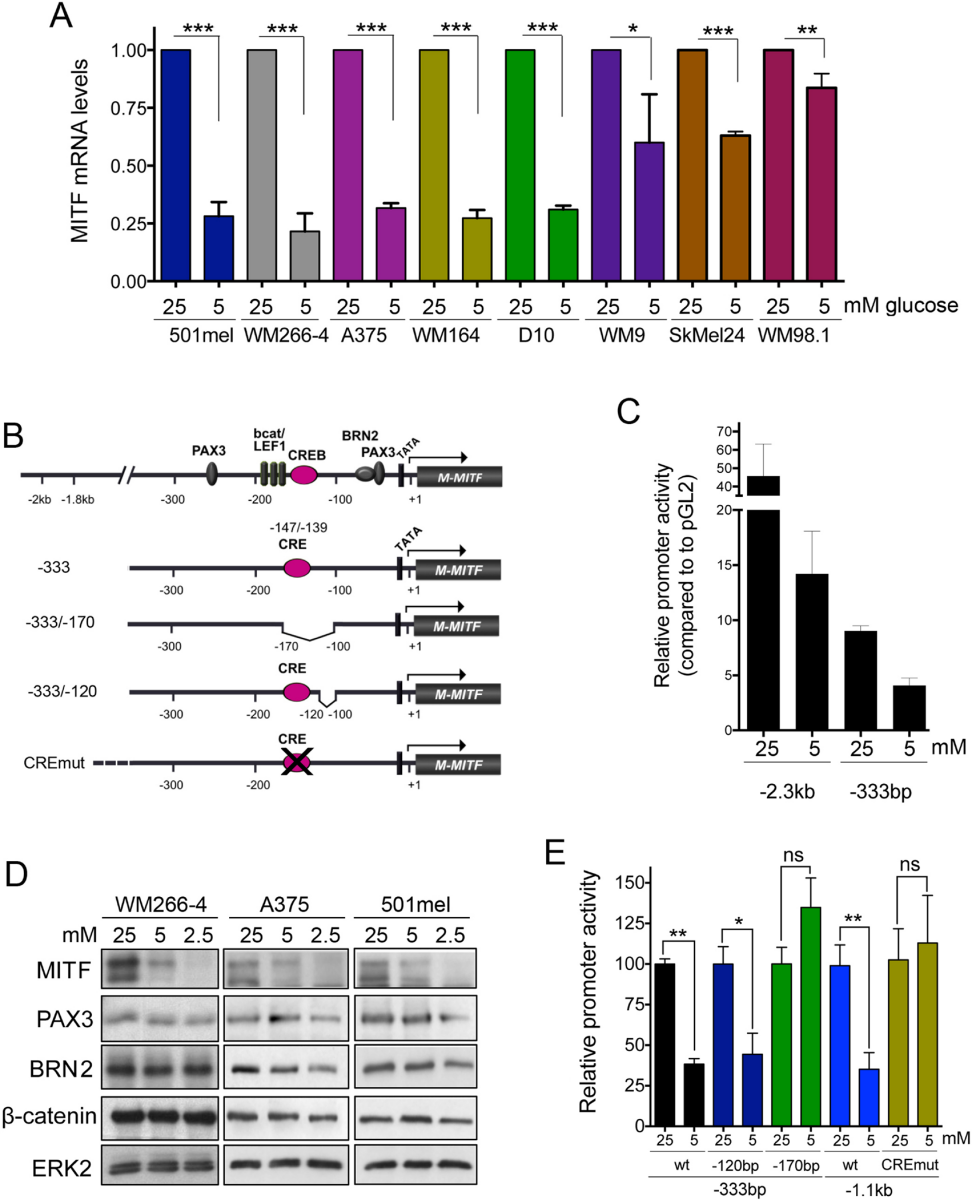
Transcriptional regulation of MITF by glucose (**A**) Relative MITF mRNA levels in a panel of eight melanoma cell lines cultured for 48 h under physiological glucose conditions (5 mM), compared to cells grown at 25 mM glucose. (**B**) Schematic of the *MITF* promoter and the most prominent melanocyte specific transcription factor binding sites. Also shown are schematics of the -333 *MITF* promoter fragments either containing (−333; −333/−120) or lacking an active CRE site (−333/−170, CREmut) (**C**) Promoter activity of the indicated *MITF* promoter fragments was measured as luciferase induction in 501mel cells cultured at 25 mM or 5 mM glucose for 48 h. Fold activation of the promoter is relative to the activity of the empty vector pGL2. (**D**) Western blot for the expression of MITF, PAX3, BRN2 and β -catenin in lysates from WM266-4, A375 and 501mel cells cultured at varying concentrations of glucose for 48 h. (**E**) The activity of the indicated *MITF* promoter fragments in 501mel cells cultured at 25 Mm or 5 Mm glucose for 48 h is shown. Fold activation of the promoter is relative to the activity of each promoter portion in cells grown at 25 mM.

The fact that MITF expression from an ectopic promoter could overcome glucose-dependence in melanoma cells (see Figure 2E), suggested that glucose regulates MITF at the transcriptional level. Indeed, in a panel of eight melanoma cell lines glucose levels regulated MITF mRNA levels (Figure 3A). Furthermore transcription from a 2.3 kb *MITF* promoter fragment containing most of the relevant regulatory sites (Figure 3B and [15] revealed that glucose deprivation reduced the promoter activity (Figure 3C). When utilising a shorter promoter fragment (-333) harbouring the major melanocyte specific regulatory sites, we obtained similar results (Figure 3C), which suggested that the site of glucose action is located within the first 333 base pairs upstream of the transcription start site. Importantly, the expression of the main transcriptional regulators active in this region, such as beta-catenin, PAX3 or BRN2 [15–18] was unchanged upon glucose restriction (Figure 3D).

When measuring the transcriptional activation of another construct, -333/−170, which lacks 70bp upstream of the BRN2 and PAX3 binding sites, promoter responsiveness to glucose levels was lost (Figure 3E). Importantly, this region contains a cAMP response element (CRE) located at -147/−139 (Figure 3B), which is a major binding site for CREB downstream of signalling from the MC1R [19, 20]. Removing 20bp downstream of the CRE did not have an effect of glucose mediated promoter regulation, which strongly suggested an involvement of the CREB binding site. Indeed, a 1.1 kb promoter construct, in which the CRE had been inactivated by point mutation was unresponsive to glucose levels compared to the wt 1.1 kb fragment (Figure 3E). This suggested that an intact CRE was crucial for glucose mediated regulation of MITF transcription.

### High glucose levels reduce ATF4 mediated suppression of MITF expression

**Figure 4:**
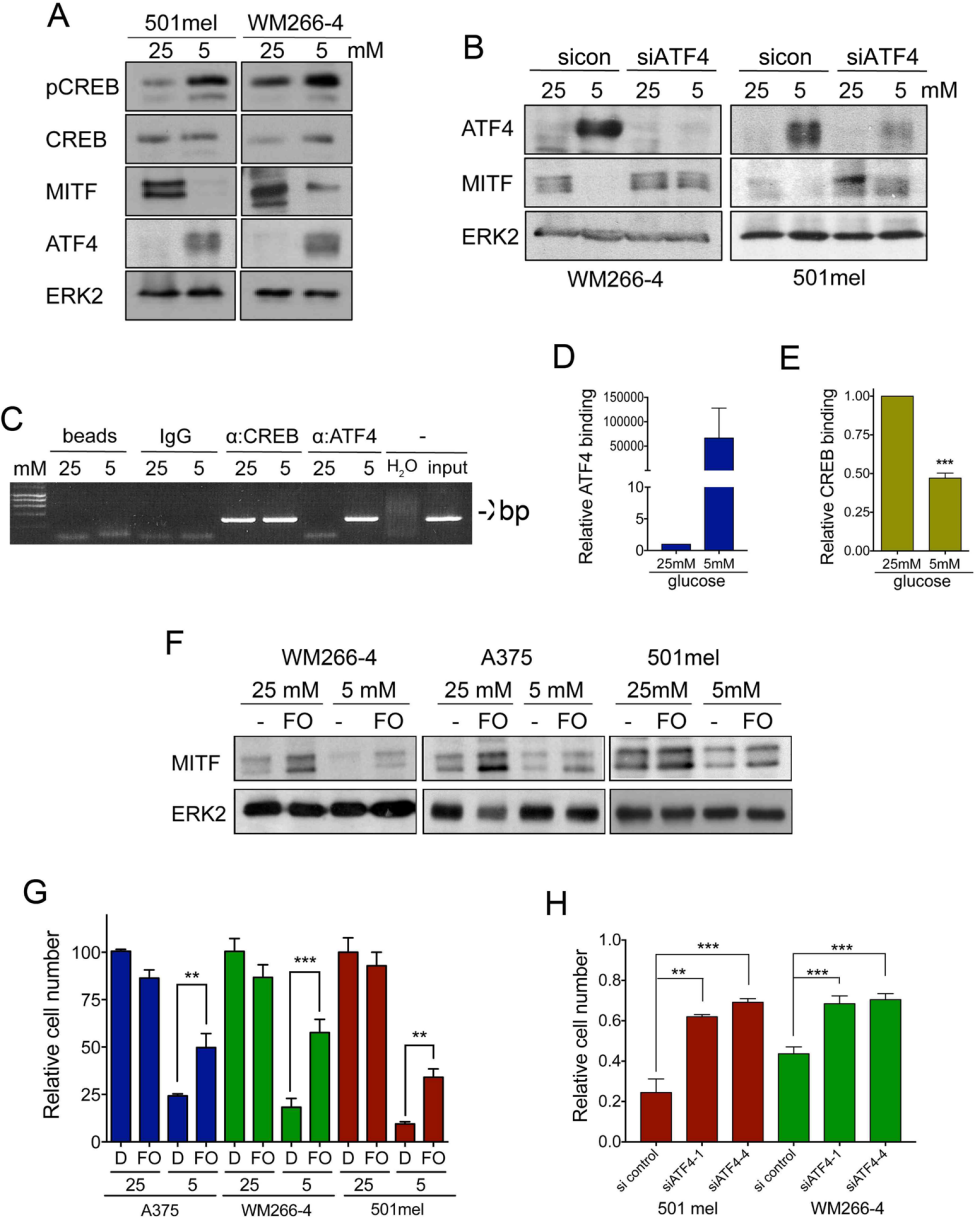
ATF4 suppresses MITF expression upon glucose restriction (**A**) Western blot for active, phospho-CREB (pCREB), total CREB (CREB), MITF and ATF4 protein levels in lysates from 501mel and WM266-4 cells cultured as indicated. ERK2 was used as a loading control. (**B**) ATF4 and MITF levels in WM266-4 cells transfected with 40nM scrambled control siRNA or an ATF4-targeting siRNA SMART pool (siATF4) for 72 h. Cells were cultured at 25 mM or 5 mM glucose for the last 48 h. ERK2 was used as loading control. (**C**) Chromatin immuno-precipitation (ChIP) assay using CREB and ATF4 specific antibodies. The region encompassing −420/−134 of the M-MITF promoter and which contains the CREB binding site was amplified by RT-PCR at saturating conditions (40 cycles). (**D**–**E**) Quantitative RT-PCR was performed in samples processed as in E to determine the relative binding of either ATF4 or CREB to the proximal CREB binding site in the MITF promoter. (**F**) Western blot for MITF in cells cultured at 25 or 5 mM glucose in the absence or presence of 20 μM forskolin (FO). (**G**) Relative growth of melanoma cells treated as in F. (**H**) Graphs show the relative cell proliferation of 501mel and WM266-4 cells transfected with 40 nM control siRNA or two individual ATF4-targeting siRNAs for 72 h, at 5 mM glucose compared to cells cultured at 25 mM transfected with control siRNA. ***p < 0.001, **p < 0.01.

We found that the CREB binding site is central to the glucose-mediated regulation of MITF expression, whereby glucose deprivation results in reduced MITF expression. Intriguingly, both CREB activation and increased expression have been linked to glucose deprivation [21, 22], and we find a similar response in melanoma cells (Figure 4A). Importantly, while CREB is a strong activator of the MITF promoter [20], we find that glucose deprivation reduces MITF expression. This suggests that another factor needs to overcome the function of CREB at the *MITF* promoter. Intriguingly, ATF4, another CREB family member, has been involved in nutrient-stress mediated gene regulation, and can act as suppressor at the CREB binding site [23]. Indeed, glucose restriction resulted in a strong up-regulation of ATF4 in melanoma cells, and this correlated with reduced MITF expression (Figure 4A). Moreover, depletion of ATF4 using RNAi technology prevented the reduction in MITF expression in response to glucose restriction (Figure 4B and Supplementary Figure 2), clearly demonstrating that ATF4 acts as the link between glucose metabolism and MITF in melanoma cells.

Using ChIP, we found that in line with its central role to regulate MITF expression, CREB binds the *MITF* promoter under both high (25 mM) and physiological (5 mM) glucose levels (Figure 4C). However, ATF4 only bound to the MITF promoter when glucose levels in melanoma cells were restricted (Figure 4C). Further quantification using qRT-PCR revealed that when ATF4 was bound to the MITF promoter, CREB binding was reduced (Figure 4D and 4E), which suggests that ATF4 competes with CREB for binding at the *MITF* promoter, thereby reducing MITF expression. Classically MITF expression is regulated by CREB in a cAMP signalling manner, and therefore the adenylate cyclase activator forskolin is a potent inducer of MITF expression [20]. Thus we made use of forskolin to activate CREB and test the interplay beween cAMP-CREB signalling and glucose restriction. Treatment of melanoma cells with forskolin was capable to almost fully restore MITF expression under glucose limitation (Figure 4F). Moreover, at 5 mM glucose, forskolin-dependent MITF re-expression was accompanied by a significant increase in cell growth (Figure 4G). To further assess whether ATF4 was acting as a repressor of melanoma cell proliferation under low glucose conditions we used RNAi to block ATF4 expression and as shown in Figure 4H, melanoma cell proliferation was significantly restored (Figure 4H). Our data strongly indicated that glucose restriction inhibits melanoma cell proliferation by inducing ATF4 to subsequently interfere with MITF expression in a CRE dependent manner.

### Glucose restriction affects glycolysis and mitochondrial respiration

**Figure 5:**
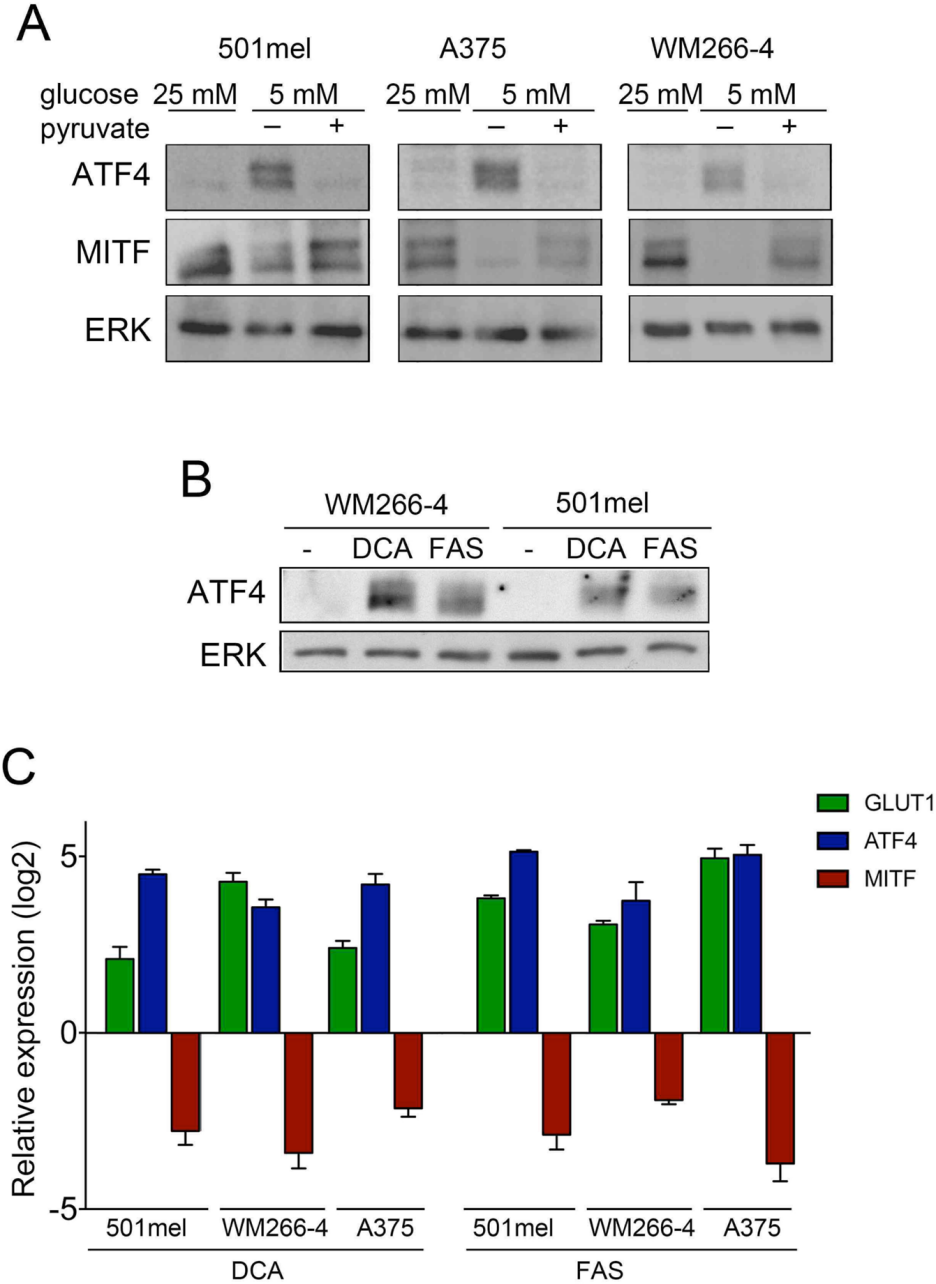
Glucose metabolism inversely regulates ATF4 and MITF (**A**) Western blot for ATF4 and MITF in cells cultured at 25 mM or 5 mM in the presence or absence of 1 mM pyruvate. (**B**) ATF4 levels in cells treated with the glycolysis inhibitor dichloroacetate (DCA, 50 mM) or Fasentin. (FAS, 72 μM) for 48 h. ERK2 was used as loading control. (**C**) Relative GLUT1, ATF4 and MITF mRNA levels in cells treated with dichloroacetate or fasentin as in B.

We next attempted to better understand how glucose restriction affects ATF4 and consequently MITF expression. When melanoma cells were cultured under 5 mM glucose and supplemented with pyruvate, a glycolysis intermediate, ATF4 expression was not induced and concomitantly, MITF expression remained unchanged (Figure 5A) suggesting that glucose limitation to physiological levels of glucose has an impact in the glycolytic capacity of melanoma cells. Similarly treatment of cells with the GLUT1 inhibitor fasentin (FAS) or the pyruvate dehydrogenase kinase inhibitor dichloroacetate (DCA) induced the expression of ATF4, while expression levels of MITF were markedly reduced (Figure 5B and 5C). These results further supported the idea that glucose restriction was affecting glycolysis. Indeed measurement of extracellular pH levels demonstrated that glucose restriction induced an alkalinisation of the culture media in a time dependent manner (Supplementary Figure 3A). Intriguingly the levels of ATP did not significantly vary after 48 h of cell culture under glucose restriction (Supplementary Figure 3B) suggesting a compensatory mechanism to sustain energy levels. Accordingly when assessing mitochondrial activity by measuring oxygen consumption we observed that glucose limitation produced a significant increase in mitochondrial respiration in all three cell lines (Supplementary Figure 3C).

### Glucose availability regulates ATF4 and MITF via ROS

Our data link glucose deprivation and the regulation of ATF4 expression with an increase in mitochondrial respiration and it is known that glucose restriction can promote mitochondrial production of reactive oxygen species (ROS) [24–26]. Furthermore oxidative stress has previously been linked to downregulation of melanocyte differentiation markers [27], therefore we decided to study if ROS could be triggering glucose restriction-mediated inhibition of melanoma proliferation in a MITF dependent manner.

**Figure 6:**
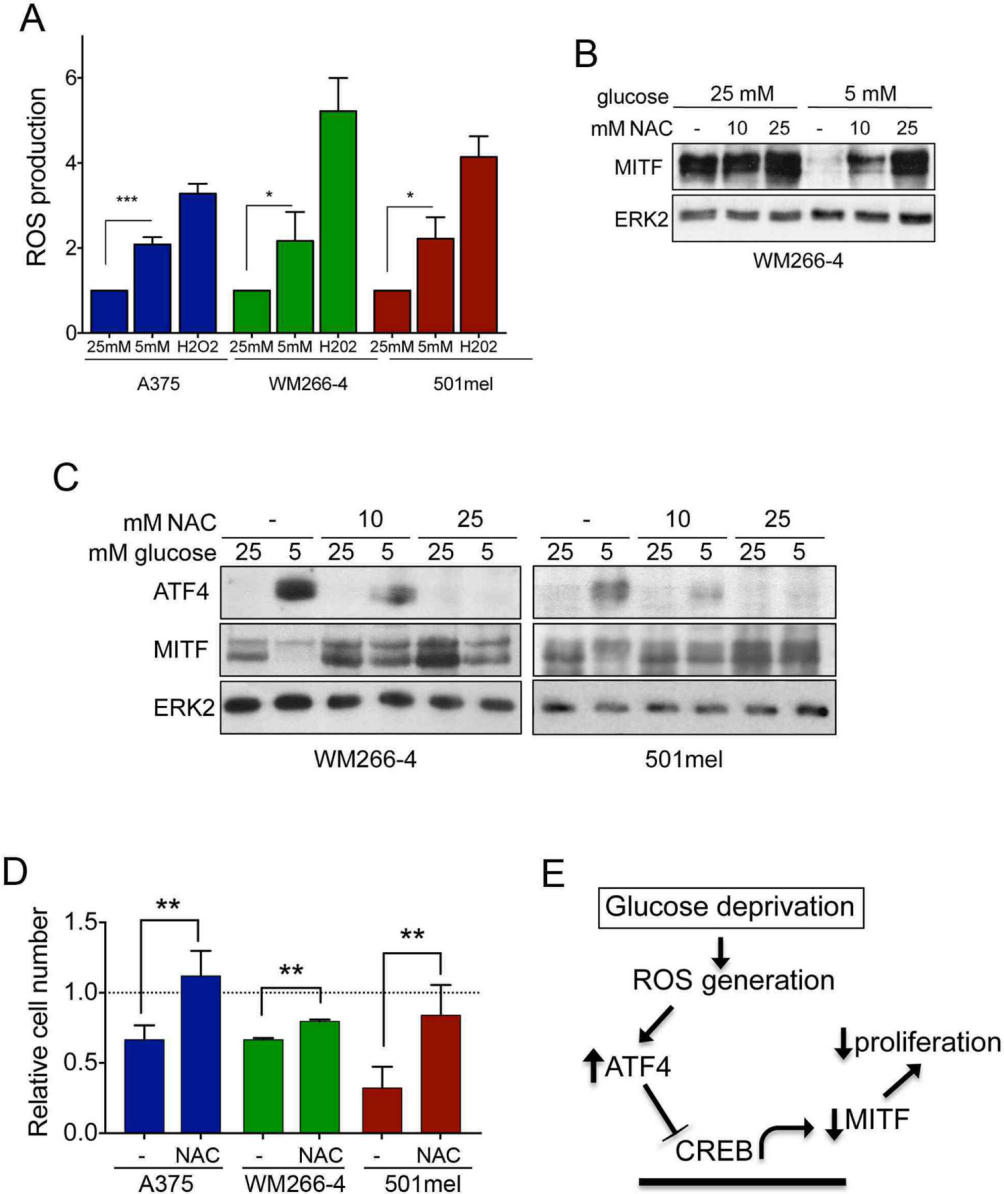
ROS production induces ATF4 expression in melanoma cells (**A**) Relatives levels of ROS in A375, WM266-4 and 501mel cells cultured for 48 h at 5 mM glucose, as compared with cells grown at 25 mM glucose. Cells treated for 30 min with 100 μM hydrogen peroxide (H202) were used as positive control. (**B**) MITF expression levels in WM266-4 cells cultured for 48 h at 25 mM or 5 mM glucose in the presence of absence of 10 mM or 25 mM of N-acetyl cysteine (NAC). (**C**) Lysates from WM266-4 and 501mel cells treated as in A, were analyzed by western-blot for the levels of ATF4 and MITF. ERK2 was used as loading control in A–C. (**D**) Relative cell numbers of A375, WM266-4 and 501mel cells cultured at 5 mM glucose either without or with 10 mM NAC for 48 h. The growth of cells at 25 mM glucose was set 1. ***p < 0.001, **p < 0.01, *p < 0.5. (**E**) Model summarizing glucose-dependent regulation of MITF

We first tested if restriction of glucose availability in melanoma cells increases ROS production. Indeed, culturing A375, WM266-4 or 501mel cells with 5 mM glucose significantly induced ROS levels (Figure 6A). In this context, ROS elevation seemed to be behind the downregulation of MITF expression, because the general ROS scavenger Sodium Acetyl Cysteine (NAC) was capable of fully rescuing MITF expression under glucose limitation (Figure 6B).

Accordingly, under 5 mM glucose NAC restored MITF expression and blocked ATF4 up-regulation in a dose dependent manner (Figure 6C). Importantly, it has been described that oxidative stress can induce the expression of HIF1alpha, which can repress MITF expression under low oxygen conditions [28, 29], but under our experimental conditions, glucose restriction did not induce HIF1alpha expression (Supplementary Figure 4).

The functional consequence of this mechanism was seen when melanoma cell growth was assessed under glucose restriction conditions, whereby ROS scavenging using NAC significantly restored cell numbers at low glucose concentrations in all three melanoma cell lines (Figure 6D)

Together, our data strongly suggest that glucose deprivation suppresses MITF expression through ROS induced ATF4 up-regulation, which in turn results in reduced melanoma cell proliferation (Figure 6E).

## DISCUSSION

The deregulated metabolism that cancer cells have adopted makes these cells dependent on the supply of metabolic substrates. In this study we have attempted to gain insight into the dependence of melanoma cells on glucose availability. We demonstrate that melanoma cells have become dependent on glucose for their growth, which is fuelled by high glucose concentrations, whereas glucose withdrawal i.e. restricting glucose to physiological concentrations reduces melanoma cell proliferation. Although the role of glucose transporters as markers of melanoma progression and the effect of BRAF inhibition on melanoma cell metabolism have been studied [4, 5, 30–35], the consequences of glucose deprivation on signalling networks key for melanoma biology have not been specifically addressed in the literature. We have determined some of the key signalling players regulating glucose stimulated melanoma cell proliferation, and provide evidence suggesting that the transcription factor MITF acts as a node between glucose metabolism and cell cycle progression.

We demonstrate that limiting the levels of glucose availability results in the expression of the transcription factor ATF4, which is a known mediator of stress pathways, including hypoxia/anoxia, nutrient deprivation and endoplasmic reticulum stress [23, 36, 37]. Although several studies have shown that ATF4 can activate transcription, it was originally identified as a transcriptional repressor binding to and acting on the CRE site [38]. Indeed, it is its suppressor function that suggests that ATF4 functions as a repressor of long-term memory storage [38, 39]. We found that in the context of glucose deprivation in melanoma, ATF4 negatively regulates the CRE of the *MITF* promoter. Under physiological conditions the CRE site is bound by CREB, which is one of the strongest transcriptional inducers of the *MITF* promoter [20]. While during alpha-MSH induced melanocyte differentiation CREB is activated through PKA downstream of the MC1R [19], we find that in melanoma cells CREB constitutively binds to the *MITF* promoter and contributes to the basal expression of MITF. On the other hand, we show that ATF4, when expressed, is able to reduce the amount of CREB binding to the *MITF* promoter. Thereby it appears to act as both, competitor of CREB and transcriptional repressor.

Interestingly glucose restriction seems to induce further hyper-activation of the MAPK pathway while reducing melanoma cell proliferation. While this seems contradictory in the first place, the explanation lies in the fact that in melanoma cells MITF is a crucial stimulator of cell cycle progression downstream of ERK [15]. This means that loss of MITF expression will affect cell cycle regulators such as CDK4, CDK2 and p27 and hence G1/S transition despite the presence of active ERK.

It is well known that in order to promote tumour formation cancer cells under oxygen stress produce pro-angiogenic factors such as VEGF to promote the formation of newly formed vessels and provide the nascent tumour with oxygen and nutrients from the blood. Previous reports have suggested that the pro-angiogenic response of melanoma to low levels of oxygen depends at least in part on HIF1α mediated down-regulation of MITF [29]. In this context it is noteworthy that glucose limitation-dependent regulation of MITF levels appear not to be linked to a hypoxic response through HIF1α, as we find that HIF1α is not upregulated under low levels of glucose. In addition, and contrary to previous reports [36], we do not observe an induction of ATF4 in melanoma cells under hypoxic conditions. Contrary to oxygen diffusion, glucose requires active transport from the blood stream to high-glucose demanding cells such as melanoma cells so it is very likely that a hypoxic microenvironment must also present the tumour with limited availability of glucose. In this regard we observe that this is might be the case, since glucose restriction promotes the induction of the regulators of angiogenesis VEGF and interleukin-8 at the mRNA level (Supplementary Figure 5A). Furthermore cells cultured at 5 mM glucose increase the levels of the tyrosine kinase receptor AXL, a marker for melanoma cells with a more invasive phenotype (Supplementary Figure 5B) [40], and indeed glucose restriction significantly increases melanoma cell invasiveness through collagen I 3D-matrices (Supplementary Figure 5C). In summary these preliminary data suggest that glucose restriction might promote a transition towards a more aggressive phenotype characterised by slow cycling, highly invasive, MITF-low cells/AXL-high melanoma cells with potentially the capacity to contribute to the early stages of the metastatic cascade by producing pro-angiogenic factors such as VEGF and interleukin-8.

In the future it will be interesting to investigate in *in vivo* settings if in melanoma glucose deprivation might contribute to the angiogenic and pro-metastatic responses triggered by low oxygen availability and whether ATF4 complements HIF1α's role in promoting the formation of new blood vessels to irrigate and feed the tumour.

We identified a role for ROS in the regulation of ATF4, and in line with previous reports, we show that glucose deprivation induced the production of reactive oxygen species (ROS) [25, 41]. ROS can react with nucleotides in DNA, which, if unrepaired, can lead to mutations. If these mutations affect critical genes such as oncogenes or tumour suppressor genes, ROS can actually impact on multiple stages of multistep carcinogenesis, but its role appears to be extremely complex [42]. In melanoma ROS can contribute to the accumulation of mutations via UV-induced mutagenicity, and there is evidence suggesting ROS production is required to promote melanoma initiation and that anti-oxidants would have a protective effect against melanoma onset [43, 44]. On the other hand, ROS has also been involved in oncogene-induced senescence in melanocytes, which can be by-passed with NAC [45]. Thus, how glucose availability impacts on oncogenic transformation of melanocytes, UV-induced melanomagenesis or oncogene-induced senescence warrants further investigation.

Similarly complex is the role of elevated ROS in melanoma progression [46]. Increased ROS levels have been shown to effectively suppress RHO-ROCK mediated rounded-amoeboid invasion [47], but on the other hand an aged microenvironment induces ROS in melanoma cells, which creates slow-growing invasive tumours with increased potency to metastasise to the lung [48]. The latter observation is in line with a ‘MITF-low’ phenotype of melanoma cells, a situation we would expect in the context of glucose deprivation. Our preliminary results would therefore support a pro-invasive role for ROS in the context of melanoma cells under glucose restriction. The possibility of an aged microenvironment modulating glucose availability to neighboring melanoma cells to affect their metastatic potential represents an attractive working hypothesis.

Could glucose-dependent ROS production in melanoma cells explain reports correlating hyperglycemia and obesity with increased melanoma risk [9–11]? In light of our data we think that in the future it will be important to investigate the contribution of glucose restriction at physiological fasting and post-prandial levels concentrations to melanoma initiation and progression (invasion, resistance to anoikis and metastatic potential). Together with recent reports suggesting that fasting cycles can significantly affect tumour growth and responses to chemotherapy [49] it will be of upmost interest to determine the role of ATF4 in all these processes and the interplay between ATF4 and the master regulator of melanoma biology MITF.

## MATERIALS AND METHODS

### Cell culture and reagents

Melanoma cell lines were cultured in Dulbecco's Modified Eagle's Medium (DMEM) (SIGMA) supplemented with 0.5% penicillin and streptomycin (SIGMA) and 10% bovine calf serum (PAA, Yeovil, UK). Cells were grown at 37°C in a 5% CO2 environment. Cell lines had been authenticated by short tandem repeat allele profiling 6 months prior to the study. Cell stocks were expanded and vials kept in liquid nitrogen. New aliquots were thawed every 5–7 weeks. Human melanocytes were from Cascade Biologics and where grown according to manufacturer´s guidelines. Medium with varying concentrations of glucose was obtained by mixing DMEM with 4.5 g/l (25 mM) or no glucose in the appropriate proportions. N-acetyl cysteine (NAC), CoCl2, dichloroacetic acid (DCA), fasentin (FAS) and hydrogen peroxide were from SIGMA.

### Proliferation assays

Cells were plated in 6-wel plates and the next day was DMEM 1%FCS with varying concentrations of glucose was added. The relative number of cells compared to day 0 was assessed every 24 h over a period of 3 days by fixing and staining as described in [50].

### Apoptosis and proliferation using IncuCyte assays

To assess apoptosis induction and proliferation, 5000 cells were seeded per well in BD Falcon black tissue culture treated 96 well plates (SLS).

After 24 h media replaced to fresh DMEM containing 25 mM or 5 mM glucose and the IncuCyte Kinetic Caspase-3/7 Apoptosis Assay Reagent (Essen BioScience) was added. Apoptotic cells can be detected by a fluorescence signal if caspase 3/7 have been activated. Cells were imaged using an IncuCyte ZOOM (Essen BioScience) under 20× lense and kept at 37°C in a humidified 5% CO2 incubator. Phase contrast and fluorescence images with two to four images per well were taken every 20 min and IncuCyte ZOOM software was utilised in real-time to measure confluency, as a proxy for proliferation, and apoptosis, respectively. IncuCyte ZOOM data and timing data were imported into Prism 6 (GraphPad) for statistical analysis and presentation.

### Western blotting

Cell lysates were prepared using RIPA buffer and analysed as described in [14]. Primary antibodies used were: MITF (Thermo Fisher Scientific), phospho-ERK (MAPK-YT) (SIGMA), ATF4 (NEB), CREB and phospho-CREB (Cell Signalling), ERK2, BRN2, beta-catenin, PAX3 and HIF1alpha (Santa Cruz Biotechnologies).

### FACS analysis

100000 cells per well were seeded in 6-well plates and cultured under 25 mM or 5 mM concentrations of glucose. After 3 days, cells were harvested, washed and fixed in 80% ice-cold ethanol in PBS and kept at −20C. Cells were then washed in PBS and incubated in a solution containing PBS, RNase A (30 μg/ml, SIGMA) and Propidium Iodide (20 μg/ml, SIGMA) at 37°C for 1 hour. In order to assess the percentage of cells in each phase of the cell cycle DNA content analysis was performed using FACS Calibur (Becton Dickinson).

### EdU incorporation assays

Cells were labelled with 10 μM 5-ethynyl-2-deoxyuridine (EdU, Thermo Fisher Scientific) for 4 h before they were formalin fixed and processed following the manufacturer's instructions. Stained cells were imaged using a BDpathway 855 Bioimager and analysed to determine the relative percentage of cells positive for EdU, indicative of the proportion of cells at the DNA-synthesis phase (S-phase) of the cell cycle.

### RNA interference and transfection

The sequence of the siRNA control or Scramble (SC) was: AAUAUAAUCACUAUCAGGUGC- siRNA against ATF4 were from GE Healthcare, Amersham. with references:

ATF4 SMARTpool L-042737-01-0005

ATF4 single J-005125-10 (A1)

ATF4 single J-005125-13 (A4)

To downregulate the expression of ATF4, 200.000 cells were seeded in 6-well plates and 24 h later the medium was replaced with serum-free DMEM. Then 200 μl of mix containing 6 μl of Lipofectamine (Thermofisher) and a final concentration of 40nM siRNA, were added drop wise to the cells and left to incubate at 37C, 5% CO2 for 6 h. Medium was then replaced with 10% FCS DMEM and cells were further cultured for 48 h before ATF4 and MITF expression was assessed.

### Luciferase reporter assays

Approximately 2.3 kb of the *MITF* promoter (−2293 to +120) and the various truncated promoters were cloned into pGL2 (Promega), and the CRE mutant −1.1 kb promoter construct was generated by PCR directed mutagenesis as described [15]. The sequence of the wild type and mutant CRE site are: CRE wild type: 5′…TTTGATAGTGACGTCAAGCCA…3′, CRE mutant: 5′…TTTGATAGCGACGTCAAGCCA…3′. Cells were transfected with 1μg of different MITF-reporter constructs and 0.5 μg of pSV-beta-galactosidase. Cultures were assessed for luciferase activity after 48 h using a Firefly Luciferase assay kit (Biotium 30003-1). The data are corrected for beta-galactosidase using the Galacto-Light Plus kit (Thermo Fisher T1007) and represent activity for assays performed in triplicate, with error bars representing standard errors from the mean.

### Chromatin immunoprecipitation

Chromatin immunoprecipitation assays, using control IgG (Santa Cruz Biotechnology) or antibodies specific for CREB (Cell Signalling, 86D10), and ATF4 (Cell Signaling D4B8) were performed as described previously (Wellbrock et al. 2008). Primers for the M-MITF promoter specific to the region of CREB binding (−420 bp to −134 bp from ATG) were GCAGTTATTCGGCCATTGGA and GGAAGCCCTACGAGTTTGGT.

### ROS measurement

Cells were cultured as indicated and then loaded with 10 μM carboxylated-H2DCFDA (Thermofisher) for 1 h. Levels of oxidised (fluorescent) intracellular dye were measured using a spectrophotometer (BIO-TEK^®^, NorthStar Scientific) according to manufacturer's instruction.

### ATP production measurement

To assess the energetic state of melanoma cells the Cell-Titer Glo Luminiscent Cell Viability Assay (Promega) was used. According to manufacturing instructions cells seeded in black walled 96-well plates were cultured under 25 or 5 mM glucose for 48 h. Equal volumes of Cell Titer Glow reagent were added to each well and plates were placed in an orbital shaker for 10min before luminescence reading were recorded using a Synergy H1M spectrophotometer.

### Oxygen consumption assay

As a measurement of mitochondrial function we utilized the Extracellular O2 consumption assay ab197243 from ABCAM. Cells were cultured in opaque transparent bottom- black 96-well plates under 25 mM or 5 mM glucose for 48 h, then Extracellular O2 consumption reagent was added to each well and samples were excited at 380 nm, fluorescent emission at 650nm was detected using a BIO-TEK^®^ H1, spectrophotometer (NorthStar Scientific). This assay is based in the capacity of oxygen to quench the Extracellular O2 consumption reagent. Increased oxygen consumption is recorded as increases in fluorescence emission.

### Invasion assays

Invasion assays analysing melanoma cell invasion into collagen (Nutacon, 2.3 mg/ml) matrices were performed as previously described [51]. In order to adjust levels of glucose in collagen matrices lyophilized DMEM (SIGMA) with glucose concentration at 1 mg/ml was reconstituted as recommended by manufacturing instructions and glucose level adjusted as required.

### Statistical analysis

Unless indicated otherwise, data are from assays performed in triplicate, with error bars to represent standard deviations or errors from the mean. Statistics used were: predominately Student *t*-test and One-way ANOVA with Dunnett's Multiple Comparison Test performed using GraphPad Prism version 4.00 for Mac OS, GraphPad Software, San Diego California USA, www.graphpad.com.

## SUPPLEMENTARY MATERIALS FIGURES



## Abbreviations

MITF: Microphtalmia associated Transcription factor
MAPK: mitogen activated protein kinase
ROS: reactive oxygen species
ATF4: Activating Transcription Factor 4
NAC: n-acetylcysteine
OXPHOS: oxidative phosphorylation
