# Supplementary material for: Glucose availability controls ATF4-mediated MITF suppression to drive melanoma cell growth
Source: Oncotarget. 2017 Mar 23;8(20):32946–59. doi: 10.18632/oncotarget.16514 (PMC5464841; doi:10.18632/oncotarget.16514)
Supplement: Supplementary file 1 [file oncotarget-08-32946-s001.pdf]

Glucose availability controls ATF4-mediated MITF suppression to drive melanoma cell growth

Supplementary Materials

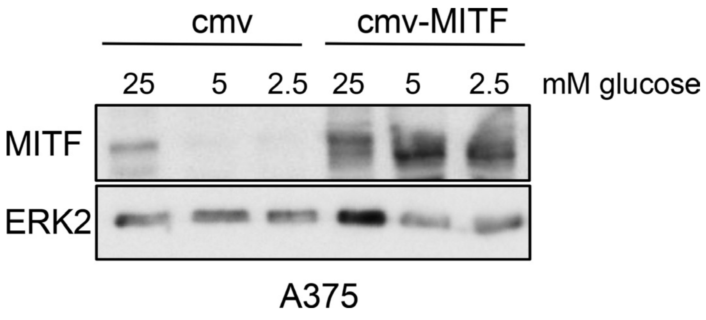

Supplementary Figure 1: MITF expression in A375 cells stably expressing an empty vector (cmv) or an MITF expression construct under the CMV promoter (cmv-MITF). Cells were cultured under varying concentrations of glucose for 48 h. ERK2 was used as loading control.

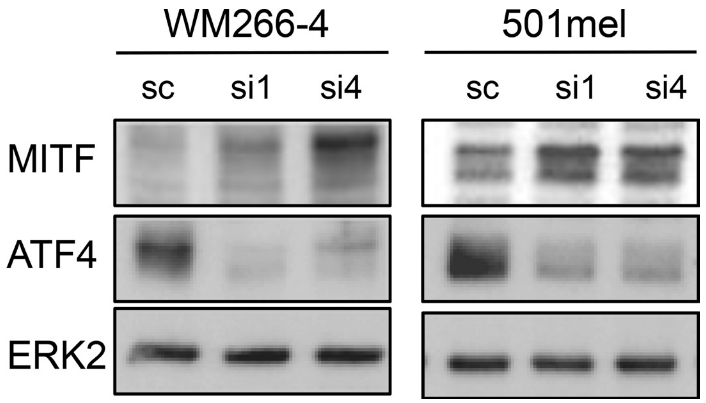

Supplementary Figure 2: ATF4 and MITF protein levels in WM266-4 and 501-mel cells transfected with scramble (sc) control or two independent siRNA against ATF4 (si1, si4) for 72 h and cultured under 5 mM glucose for the last 48 h. ERK2 serves as loading control.

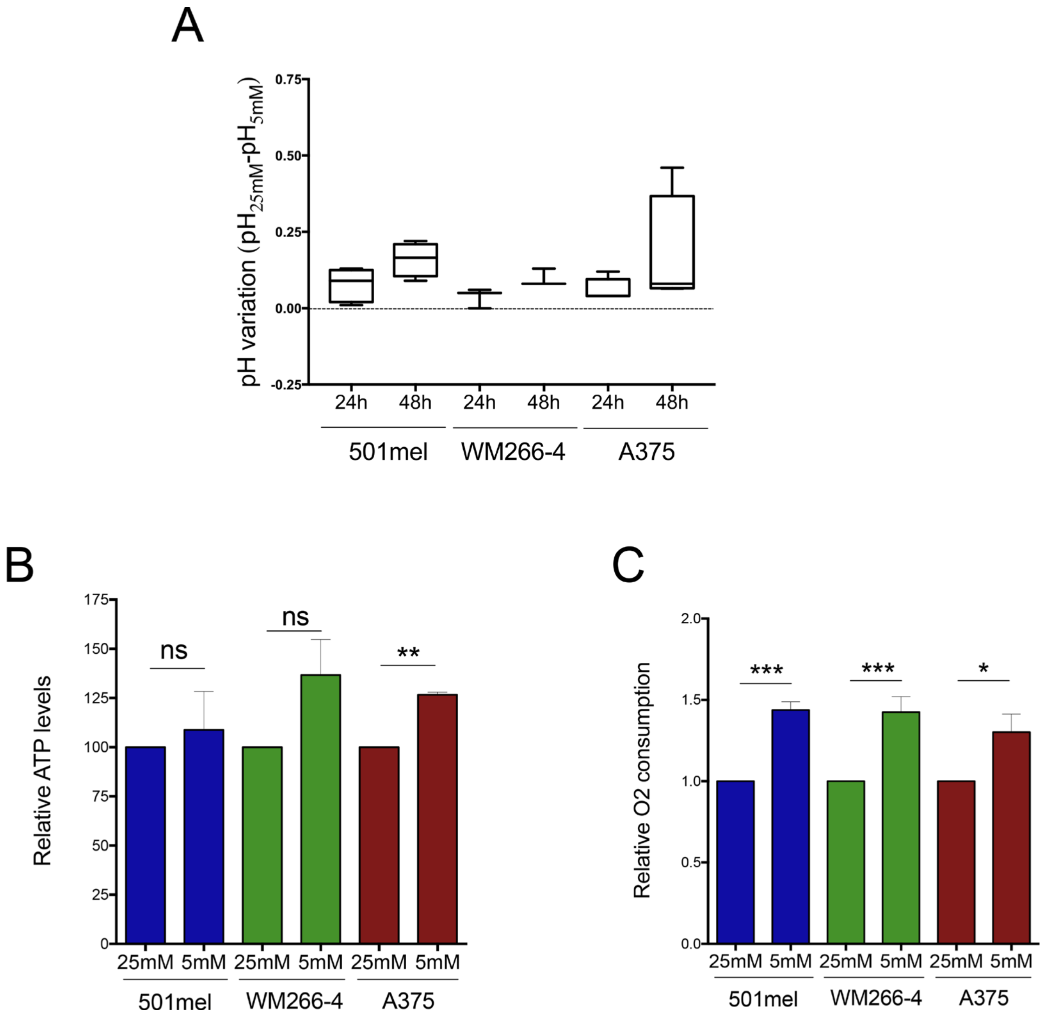

Supplementary Figure 3: (**A**) Values represent pH variations in three different melanoma cell lines between cells cultured under 5 mM glucose compared to cells cultured under 25 mM glucose over 24 or 48 h. (**B**) Relative ATP levels of melanoma cells cultured for 48 h under 5 mM glucose compared to cells cultured under 25 mM glucose. (**C**) Relative oxygen (O2) consumption levels between cells cultured under 25 or 5 mM glucose. ***$p < 0.001$, **$p < 0.01$, *$p < 0.5$, n.s.: not significant.

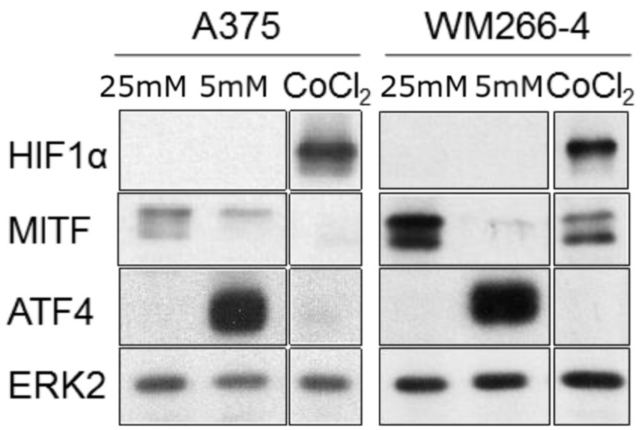

Supplementary Figure 4: MITF, ATF4 and HIF1α protein levels in A375 and WM266-4 cells treated under 25 mM or 5 mM glucose conditions or under CoCl2, 0.2 mM for 48 h. ERK2 serves as loading control.

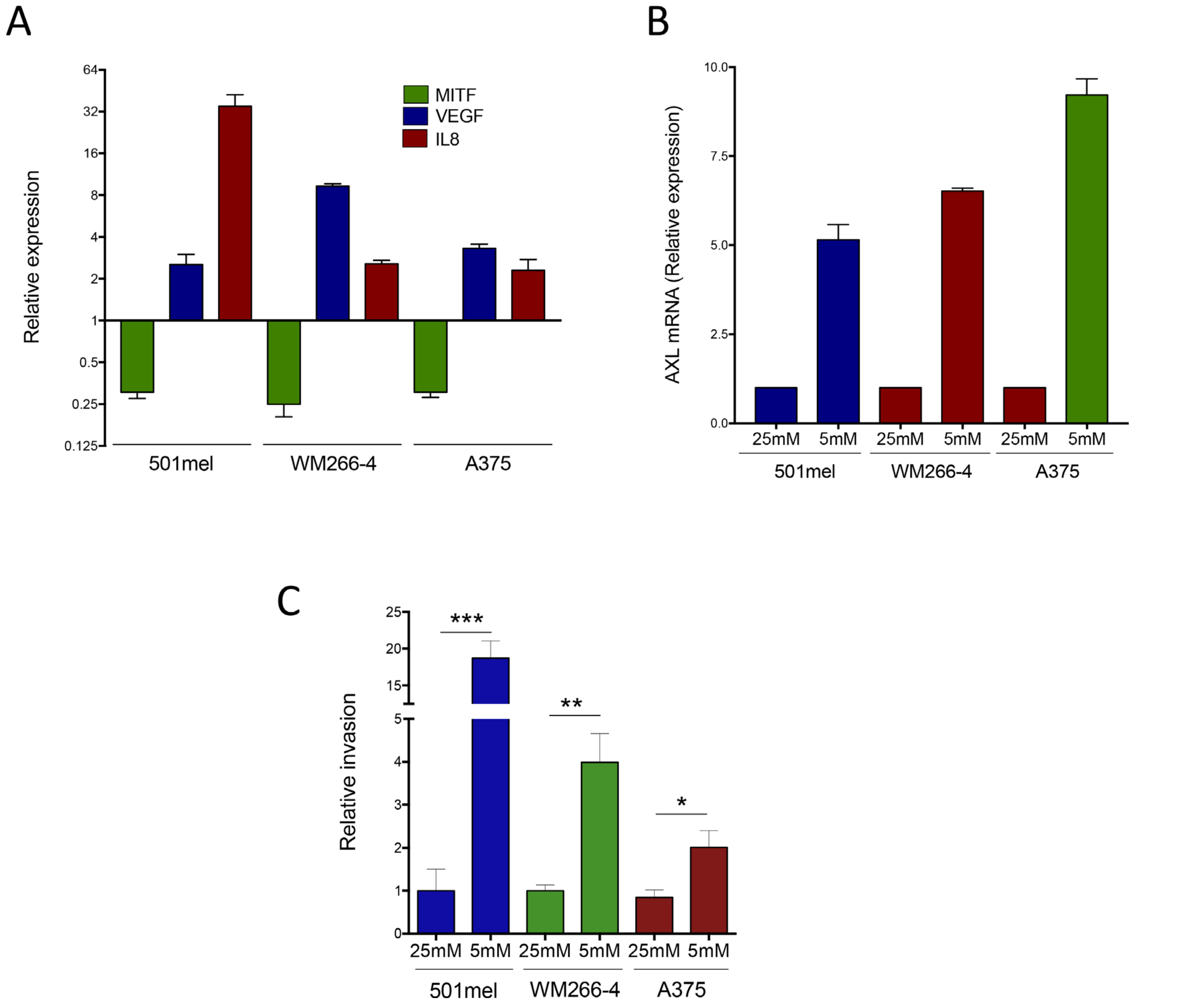

Supplementary Figure 5: (**A**) Relative MITF, VEGF and IL8 mRNA levels of melanoma cells cultured for 48 h t 5 mM glucose compated to cells treated with 25 mM glucose. (**B**) Relative AXL mRNA levels of cells treated under 5 mM. (**C**) Fold invasion in collagen I matrices containing 5 mM glucose as compared to cells invading matrices with 25 mM glucose.
